# Global Pregnancy Collaboration symposium: Prepregnancy and very early pregnancy antecedents of adverse pregnancy outcomes: Overview and recommendations

**DOI:** 10.1016/j.placenta.2017.07.012

**Published:** 2017-12

**Authors:** James M. Roberts, Christopher W.G. Redman, for the Global Pregnancy Collaboration

**Affiliations:** a Magee-Womens Research Institute, Department of Obstetrics Gynecology and Reproductive Sciences, Epidemiology and Clinical and Translational Research University of Pittsburgh, Pittsburgh, PA, USA; b Nuffield Department of Obstetrics and Gynaecology, University of Oxford, John Radcliffe Hospital, Oxford, OX3 9DU, UK

## 1 Introduction

It is becoming increasingly evident that the origins of adverse pregnancy outcomes occur at a far earlier time in gestation than has usually been considered in pathophysiological studies of these conditions. To date most studies have addressed changes occurring at 10–20 weeks menstrual age, the time of vascular remodeling of the spiral arteries supplying the intervillus space. This is stimulated by the common pathophysiological changes identified in these vessels in preeclampsia, fetal growth restriction, preterm birth, abruption and stillbirth. The changes also provide a plausible basis for the generation of the placental and systemic changes in these disorders.

However, it is clear that many important changes occur prior to this time. Epidemiological studies and studies of decidua support prepregnancy changes predisposing to abnormal pregnancy outcome. Furthermore, the few interventions to prevent adverse pregnancy outcomes that are effective seem to be most successful when given prior to or very early in pregnancy (e.g. folic acid). Likewise nutritional supplements such as calcium given during pregnancy are never as effective as lifelong dietary maintenance of adequate intake.

Guided by these concepts the Global Pregnancy Collaboration organized a workshop, “Prepregnancy and very early pregnancy antecedents of adverse pregnancy outcomes,” in September of 2016. The goal was to identify research areas that might enable the determination of mechanisms for currently recognized associations of prepregnancy and early pregnancy events with abnormal pregnancies. The ultimate goal was to identify potential targets for timely interventions.

Presentations were divided into prepregnancy and early pregnancy events that might provide such mechanisms and potential targets. Manuscripts are presented on the several topics addressed. The goal of this presentation is to provide an overview and summarize recommendations presented in the manuscripts or in the discussion stimulated by them.

## 2 Prepregnancy changes

Several prepregnancy events are well established as associated with abnormal pregnancy outcomes. Many have been recognized epidemiologically and others by pathological and pathophysiological findings. Some are recent while others have long been recognized but largely ignored. The workshop considered these epidemiological findings, especially nutritional, but also considered the role of the prepregnancy and early pregnancy decidua and the fascinating topic of microchimerism, which may have an impact before conception or in early pregnancy.

### 2.1 What are the metabolic precursors that increase the risk of preeclampsia and how could these be investigated further?

Dr. Jenny Myers addressed several of these well-recognized epidemiological associations. Wherever possible she identified mechanistic explanations for these relationships and suggested future therapeutic targets. In identifying targets with potential impact it is important to differentiate the magnitude of increased risk from proportion of preeclampsia for which this accounts. For example, although women with chronic hypertension have a 5 fold increased risk of preeclampsia compared to women without this risk factor, chronic hypertension accounts for less that 10% of preeclampsia cases. By contrast, obesity because of its high prevalence worldwide despite increasing risk only 3 fold, accounts for almost 30% of cases.

Likely for this reason, the potentially modifiable risk factor, obesity, has received the most attention directed at mechanisms and could serve as a model for the study of other epidemiologically identified risks. Obesity as determined by BMI is associated with an increasing incidence of preeclampsia as BMI increases. Although the association is strongest with late onset preeclampsia and modified by race, both variants of preeclampsia are increased in all races with increasing BMI. The mechanisms accounting for this likely reflect those that account for the increase in cardiovascular disease with obesity. Thus, features of the metabolic syndrome, inflammation and oxidative stress, are all considered relevant. The endogenous NOS completive inhibitor, asymmetric dimethyl arginine (ADMA), is increased in women with preeclampsia and further increased in women with obesity. These higher concentrations are present in early pregnancy and treatment with the arginine precursor citrulline reduces blood pressure in obese pregnant women. Angiogenic and antiangiogenic factors are also relevant, with PlGF lower in obese pregnant women and more strongly associated with preeclampsia especially of late onset.

A valuable research target is to identify differences in the 10% of obese women who develop preeclampsia. Encouraging studies have indicated dissimilar metabolic responses in subsets of obese individuals identified as metabolically unhealthy defined by abnormal hepatic lipid storage. Individuals with abnormal storage manifest metabolically abnormal responses to weight gain, which may be highly relevant for pregnancy. Similar assessments of different metabolic status in women beginning pregnancy could perhaps identify the obese woman at increased risk for preeclampsia.

Although chronic hypertension identifies a group of women with very high risk for preeclampsia there is very little mechanistic research on this relationship. This is probably explained by the multiple causes of hypertension, which discourage attempts to find a common cause. Chronic hypertension is most frequently associated with early onset preeclampsia with growth restriction suggesting abnormal implantation as an underling mechanism. In view of the high risk of chronic hypertension for preeclampsia and the increasing frequency of hypertension as women experience pregnancy at older age, exploration of the pathway to this abnormality in different types of chronic hypertension is an important topic for future investigation.

The relationship between nulliparity, the major attributable risk for preeclampsia, has been posited as due to immunological interactions related to minimal exposure to paternal antigen prior to conception. Other relationships: increased preeclampsia with a short duration of intercourse prior to conception, with new fathers and increased duration of interpregnancy internal and the use of barrier contraception support this immunological concept. Very relevant is the increased rate of preeclampsia with assisted reproductive technology. This latter relationship provides the possibility of addressing the immunological relationship in some detail by comparing techniques, which provide differential exposure to eggs, sperm and embryo. These relationships should be compared and will require collaborative studies.

It is also clear that another minimally explored area that could be enlightening is interaction of these several factors with each other and with ethnic and socioeconomic factors with especial attention to low resource settings.

### 2.2 Periconceptional nutrition and programming: new opportunities for research and patient care

Regine Steegers-Theunissen addressed nutrition as a periconceptional influence on pregnancy outcome. There is abundant evidence, experimental and epidemiological, on the relevance of appropriate and inappropriate nutrition to pregnancy. Professor Steegers-Theunissen emphasized emerging evidence of the vital role of periconceptional nutrition, from 14 weeks before to the end of the first 10 weeks after conception, on reproductive and pregnancy outcome. Interestingly and importantly, this effect is from both paternal and maternal nutrition. Inappropriate nutrition affects both the ability to conceive and the development of the fetus after conception. It is now evident that the size of the embryo is strongly related to adequate intake of appropriate nutrients in the periconceptional period. Embryo size is closely related to birth weight and thus to both acute and later life outcome for the infant. She also addressed the importance of behavioral factors, (e.g. smoking and alcohol) on fertility and outcome in this same time period. The importance of this time period targets an important period for intervention.

Diet is important not just quantitatively but qualitatively. The specific importance of micronutrients such as folic acid is well established. Less well recognized is the finding that the usual Western diet, deficient in fruits and vegetables and with excessive carbohydrates, saturated fats and animal proteins, in combination with other adverse life style patterns of men and women is associated with subfertility and adverse pregnancy outcome. It is also likely that obesity may impart its adverse impact on pregnancy outcome as a marker of a quantitatively poor diet. Conversely adherence to a Mediterranean diet, rich in vegetables, olive oil and fish is associated with increased fertility and sperm count and improved pregnancy outcome.

Professor Steegers-Theunissen and her colleagues have established a web-based mHealth application (www.slimmerzwanger.nl) directed at modifying nutrition and behavioral events in the periconceptional period. The platform assesses risk based on these factors and then uses several strategies to modify relevant behavior. In a large survey in the Netherlands, this mHealth coaching program was successful in improving qualitative nutrient intake and reducing smoking and alcohol intake. Moreover, first clinical effects indicate increased pregnancy rates for infertile and subfertile couples. This mHealth tool has been translated in an English version (www.smarterpregnancy.co.uk/research) that has been piloted in London and is now being tested in a randomized controlled trial in Southampton. The Dutch version is currently being tested in the preconceptional period in the general population, obese women and couples undergoing IVF treatment.

Thus the periconceptional period provides a potential time period to target interventions to improve pregnancy outcomes. Extending current studies to establish that these modifications will improve pregnancy outcomes is the current research goal. In addition, smart phones and the Internet are available world wide including in LMIC and this strategy should also be modified and tested in this setting.

### 2.3 Emerging role for dysregulated decidualization in the genesis of preeclampsia

Professor Kirk Conrad addressed the interesting possibility that decidual abnormalities might explain the pathophysiological placental vascular anatomical changes associated with preeclampsia and other adverse outcomes. Although these changes have provided a focus for hypotheses relevant to these disorders he presents two important disclaimers. As his title implies he proposes that the study of solely trophoblast functional abnormalities as driving this pathophysiology ignores the potential of the decidua in directing these disorders including influencing decidual immune cell and trophoblast function. He also addresses the limitation of examining term placenta and placental site biopsies and provides examples showing little, if any overlap of differentially expressed genes in trophoblast and decidua obtained at delivery from women with preeclampsia and in chorionic villus samples (CVS) obtained in early gestation in women who later develop preeclampsia.

Further evidence of the effect of decidua is the finding that differences of cytotrophoblast gene expression in cells from preeclamptic pregnancies may be reversible when these cells are removed from the uterine environment (although this could also result from the various “toxic” substances circulating during disease that not only injure endothelium, but also endometrium). The strong maternal inheritance of preeclampsia is also consistent with a role for decidua. Additionally, decidual differentiation and maturation begins prior to implantation providing a potential explanation for prepregnancy factors known to increase the risk of preeclampsia.

There are striking differences in the expression of inflammatory markers in decidua taken from women with preeclampsia. However in keeping with Professor Conrad's caution about the use of samples at the time of disease to understand preeclampsia he emphasizes that what is seen may not be cause but rather an effect of preeclampsia.

He then reviews his own data generated with chorionic villus samples (CVS) samples from women who later did or did not develop preeclampsia. Despite the small sample size, leave-one-out-cross-validation yielded 100% correct classifications and hierarchical clustering also revealed sample similarity. He reconciles his data with prior studies of endometrial maturation during the late secretory phase, and decidual maturation during intrauterine and ectopic pregnancies, from which he concludes that there is insufficient or defective maturation of decidua in women who developed preeclampsia and that the molecular signature of aberrant decidualization in CVS from women who developed preeclampsia may be independent of extravillous trophoblast. Based upon these data he provides a decidual based model for the genesis of preeclampsia and related disorders.

He argues that perhaps the only way to define the early changes in preeclampsia and related disorders is by examining decidua and trophoblast before disease onset. This is now especially problematic because chorionic villus sampling for prenatal genetic diagnosis is being replaced by non-invasive approaches. He discusses alternative strategies all of which have limitations and all of which like CVS studies demand extensive collaboration and sharing of biosamples and data.

He raises an optimistic possibility supported by reduced concentrations of IGFBP1 in early pregnancies of women with later preeclampsia. IGFBP1 is a major product of decidua and expression is reduced in CVS from women with later preeclampsia. He proposes that genes demonstrated as differentially expressed in early decidual samples from women with preeclampsia might result in diagnostic concentrations of the gene product in blood.

### 2.4 Microchimerism: defining and redefining the prepregnancy context

Dr. Hilary Gammill explored the emerging, fascinating phenomenon of microchimerism and its possible role in normal and abnormal pregnancy. Microchimerism is the long-term presence of a small number of foreign cells in an individual. In the context of this paper we discussed feto-maternal microchimerism. Women who are, or have been, pregnant carry cells from their own mothers and their fetuses. During pregnancy maternal cells traffic to the fetus where they persist into adult life. It is a two-way traffic because a pregnant woman can also acquire cells from her fetus, which interact with her own cells, those of prior fetuses and those of her mother and even cells persisting from earlier generations.

Many of these cells are of immune origin, which may affect lifelong health. Animal studies have shown the relevance of maternal microchimerism. Fetal mice acquire life-long tolerance to antigens, to which they are exposed in utero. This tolerance has also been demonstrated in humans. The long term natural consequences are unclear but are relevant to transplantation outcomes and likely relevant to next generation pregnancy outcomes.

Pregnancy and in particular abnormal pregnancy are associated with later life diseases, in ways that include microchimerism. During pregnancy for example women increasingly have detectable circulating microchimerism from their own mothers, which does not occur in women with preeclampsia. Further with increasing numbers of pregnancies maternal microchimerism is reduced. These unexplained changes raise questions about not only the relevance of microchimerism to later life health but to pregnancy outcome.

Although these questions are defined, if not answered, in this presentation, there is, in addition, a hint at evolutionary significance from studies of malaria and microchimerism. The relationship is complex. Cord bloods from women with pregnancies with placental malaria are more likely to manifest maternal microchimerism. In later life, maternal microchimerism in children is associated with positive evidence of malaria in blood. However, there is a substantial reduction in symptomatic inflammatory malaria with this microchimerism. Dr. Gammill and her colleagues speculate that this positive value of microchimerism with malaria and perhaps other infections with placental involvement, may also drive the relationship to non-infectious placentally mediated complications such as preeclampsia, preterm birth and fetal growth restriction.

At this stage there are far more questions than answers. These are presented in detail in the attached manuscript and represent an important avenue of biological communication between genetically dissimilar individuals, which may have impact on the immunological adaptation to pregnancy and its deregulation with abnormal pregnancy.

## 3 Changes in very early pregnancy

### 3.1 Early placental development and the origins of pregnancy disorders

Professor Graham Burton discussed very early placental development. He posited that abnormal formation of the cytotrophoblast shell could contribute to the pathophysiology of preeclampsia, fetal growth restriction, miscarriage, premature birth and premature rupture of fetal membranes. These complications, although related epidemiologically, are difficult to explain by a hypothesis based only on failed maternal spiral artery remodeling. He suggests that abnormalities in the formation of the cytotrophoblast shell in very early gestation (before 6–8 weeks of gestation) could provide a unifying hypothesis.

The cytotrophoblastic shell consists of a layer of cytotrophoblast 5–10 cells thick completely surrounding the conceptus and forming an interface with maternal tissues. It is formed by cytotrophoblast cells from the distal end of cell columns, which spread laterally and merge with cells from adjacent columns. The endovascular trophoblast involved in the plugging of the spiral arteries and also the interstitial trophoblast proposed to stimulate remodeling of these vessels, are derived from the maternal surface of the shell. Plugging is most prominent in the center of the developing placenta and less on the periphery where the increased perfusion results in oxidative stress leading to villus regression and the physiological process of the formation of the smooth membranes. The shell is also responsible for the adhesion of fetal and maternal tissues.

The normal formation of the shell requires rapid cellular proliferation with critical temporal and geographic precision. If this does not occur appropriately the result is a spectrum of abnormalities with miscarriage as most severe. The pathophysiological mechanisms include incomplete plugging of the spiral arteries with inappropriate, excess perfusion, failed spiral artery remodeling, and inadequate adhesion with local hemorrhage leading to oxidative stress. The latter process is especially relevant to premature rupture of the membranes and premature labor, which are proposed to occur in response to oxidative stress.

Support and regulation of the formation of the cytotrophoblast shell require interaction of maternal and fetal tissues. Rapid cytotrophoblast proliferation is maintained by nutrients provided by histotrophic nutrition from maternal endometrial glands. These glands also release stimulatory growth factors and release of these factors is activated by pregnancy hormones.

The hypothesis proposing normal formation, structure and function of the cytotrophoblast shell unifies the pathophysiology of several epidemiologically related pregnancy complications. Normal pregnancy requires appropriate formation, timing and support of the shell. Professor Burton proposes that the pre-pregnant endometrium must be appropriately prepared for this function, and that an appropriate signaling dialogue between endometrium and trophoblast is mandatory to avoid abnormal pregnancy outcomes.

### 3.2 Maternal effector T cells within decidua: the adaptive immune response to pregnancy?

Professor Paul Moss emphasized the potential importance of decidual CD4 and CD8 cells in human pregnancy. The human immune response plays a critical role in determining pregnancy outcome. For many years most attention has been directed to decidual natural killer (dNK) cells and their interaction with trophoblast HLA antigens in the regulation of trophoblast invasion. Interestingly, although dNK cells are the prominent maternal decidual immune cell in early gestation as term gestation approaches the number of decidual effector T cells come to constitute 60% of immune cells in decidua. These cells are highly differentiated; express a wide range of cytokines and a high level of genes involved in interferon signaling. Nonetheless, they have not received a great deal of attention.

Professor Moss discussed the regulation of these effector cells by regulatory T cells and emphasized the potential role of these cells in combatting local infection. However, it is also evident that these cells recognize fetal tissues. These CD8^+^ cells can be demonstrated in decidua and in the maternal circulation increasing with increased numbers of pregnancies. This is consistent with a role for such cells to regulate the fetal microchimerism described by Dr. Gammill, which decreases with increasing pregnancies.

Does the interaction of effector decidual T cells with fetal tissue have a role in abnormal pregnancy outcomes? There is little evidence of their involvement in preeclampsia, perhaps reflecting the role of these cells in later pregnancy after the establishment of the physiologically transformed spiral arteries. However, decidual T-cells have been strongly implicated in the development of chronic villitis that has been associated with fetal growth restriction and preterm birth.

It is apparent that maternal effector T cells are worthy of further investigation to more definitively determine their role to support pregnancy and to contribute to adverse pregnancy outcomes. Understanding the unique functional properties of these cells could provide novel immunotherapeutic opportunities.

### 3.3 How do HLA-KIR associations regulate human reproductive success?

Dr. Andrew Sharkey presented work summarizing two recent publications [1], [2]. His group's work has pursued the hypothesis that recognition of fetal extravillous trophoblast cells (EVT) by maternal uterine NK cells in the decidua regulates the extent of EVT invasion and vascular remodeling. Inadequate vascular conversion leading to increased risk of preeclampsia (PE) or fetal growth restriction (FGR) occurs when this uNK/EVT interaction is suboptimal. Conversely, excessive invasion and remodeling may lead to increased fetal growth and obstetric complications such as obstructed labor.

EVT express a unique array of HLA class I molecules: HLA-E, HLA-G and HLA-C. Uterine NK cells comprise up to 70% of maternal leukocytes in decidua and express relevant receptors (NKG2A, LILRB1 and members of the KIR family) for each of these HLA ligands. Maternal KIR/fetal HLA-C interactions are of particular interest because HLA-C is the only polymorphic MHC on EVT, and both maternal and paternal HLA-C allotypes are expressed. KIR are also highly variable between individuals, both in the total number of activating or inhibitory KIR genes, allelic diversity at individual KIR loci and differences in copy number. So each pregnancy is characterized by different combinations of maternal KIR and fetal HLA-C genes, resulting in variable uNK inhibition or activation.

Despite the extreme variation of KIR, a broad distinction into two basic haplotypes has been informative in epidemiological studies. The *KIR A* haplotype has fewer genes with mainly inhibitory *KIR*, whereas *KIR B* haplotypes contain additional activating *KIR*. All HLA-C alleles can be assigned to C1 or C2 groups based on a dimorphism at position 80 of the α1 domain. All individuals therefore have C1 and/or C2 epitopes that bind KIR.

The group's genetic studies show that mothers with two *KIR A* haplotypes (*KIR AA* genotype) are at increased risk of disorders of placentation if the fetus carries a C2 epitope inherited from the father. Conversely, mothers with a *KIR B* haplotype (containing activating KIR2DS1 that can also bind C2) are at low risk, but instead these mothers have an increased risk of delivering a large baby [1]. When the fetus is C1 homozygous the mother's *KIR* genotype has no effect, so C2 is the crucial fetal ligand. In addition, in pregnancies with a C2 fetus, *KIRAA* mothers who lack C2 (i.e. C1/C1) are at greater risk than C1/C2 mothers. Three conclusions emerge from the genetics:

i) Binding of the maternal inhibitory KIR2DL1 to trophoblast C2 increases the risk of pregnancy disorders, whereas activating KIR2DS1 promotes fetal growth.
ii) The interaction between inhibitory KIR2DL3 and fetal C1 appears to have no significant effect on pregnancy outcome, emphasizing the importance of KIR2DL1 and fetal C2.
iii) Maternal HLA-C status also affects pregnancy outcome, when the fetus is C2.

Overall their results suggest that receptor/ligand interactions leading to strong uNK inhibition result in decreased trophoblast invasion because this combination inhibits cytokine secretion by maternal uNK. These pregnancies are associated with increased risk of PE or FGR. In contrast, mothers with the activating KIR2DS1 gene and a C2 fetus are more likely to have large babies [2]. They have shown that when KIR2DS1 on uNK binds to cells expressing the HLA-C2 epitope, this increases secretion of cytokines that enhance trophoblast invasion.

The combination of maternal *KIRAA* genotype and fetal *HLA-C2* associated with increased risk of PE and FGR is present at 12% of UK pregnancies, while only 4–5% of pregnancies develop PE or FGR. One explanation is that within the *KIRAA* genotype there are high and low risk KIR2DL1 alleles that bind HLA-C2 to different extents. Genetic studies are underway to define which specific KIR2DL1 and HLA-C allele combinations confer the greatest risk of inadequate vascular conversion and hence preeclampsia or FGR.

### 3.4 Strategies for investigating the maternal-fetal interface in the first trimester of pregnancy: what can we learn about pathology?

Professor Judith Cartwright addressed the importance and challenges of studying very early changes occurring at the maternal fetal interface. She then described a strategy her group has used to address this challenge. The complexity of maternal fetal interaction involves the interplay of multiple cell types. The remodeling of the maternal spiral arteries to the high volume low resistance status necessary for normal pregnancy involves endovascular trophoblast (EVT) affecting vascular cells. However, the activity/function is influenced by maternal immune cells in the decidua, which themselves seem to directly influence vascular remodeling. Successful remodeling is not solely or inevitably a manifestation of normal invasion. Plugs of EVT can be found in vessels that have not remodeled emphasizing the importance of functional assessment.

It is challenging to study these very early interactions. Animal studies have value but are limited by differences in invasion (e.g. shallow versus deep invasion) in different species. Professor Cartwright points out the problems of extrapolating findings at the end of pregnancy to the relevant functions in the first trimester. She also agrees with Professor Conrad both on the power of CVS and the increasing rarity of these samples. They are also limited by the small amount of tissue that they yield. Studies of cells isolated from early pregnancies can reveal cellular mechanisms but cannot discriminate normal from abnormal findings because late pregnancy outcomes cannot be known.

As a proxy for late outcome her group uses uterine arterial Doppler velocimetry, assessed prior to termination of pregnancy. The samples can then be assigned to two groups coming from pregnancies at higher and lower risk of adverse late pregnancy outcomes. Morphological findings and *in vitro* cellular function are compared in the two groups. Although this must be a relatively blunt tool since most pregnancies with abnormal findings are known to have normal outcomes, the 5 fold increased risk is associated with very different cellular findings from those in samples from individuals predicted to have normal pregnancy outcomes.

Morphological assessment of tissues from high-risk pregnancies indicates abnormal invasion of EVT with fewer vascular plugs. The ability to separate numerous cell types in these samples has allowed them to demonstrate functional differences in EVT, and decidual natural killer cells including interaction of these cell types. Early information about mechanisms involved in this disordered function is being obtained including different expression of receptors and cytokines relevant to these functions.

Professor Cartwright admits the limitations of Doppler assessment to determine future pregnancy outcome. Future strategies are described to strengthen this association using other markers (e.g. in blood) of adverse outcome. This approach has the additional strength of perhaps identifying subtypes of pregnancies determined as higher risk by Doppler assessment, which might indicate mechanisms by which certain pregnancies overcome functional abnormalities. Better approaches to cell separation and more “physiological” assessment of cell function and multicellular interactions *in vitro* with advanced cell culture techniques currently used in cancer research offer great promise. Even with present techniques more detailed mechanistic assessment is possible with studies relating genes, microRNAs, proteome and secretome with functional findings. The ultimate goal is therapy and this system will allow *in vitro* assessment of manipulations to normalize function.

## 4 Overview

In the concluding session Professor Christopher Redman overviewed the issues raised during the meeting. He also pointed out important implications of these findings and additional considerations relevant to prepregnancy and early pregnancy genesis of abnormal outcomes.

Poor placentation is a major precursor to early onset preeclampsia, usually associated with fetal growth restriction. Professor Burton proposed an important new view of placentation, in which deficient spiral artery remodeling is not a primary pathology but the end-stage of a much earlier, tightly coordinated and precisely timed sequence of placental development. The pathology centers on underdevelopment of the cytotrophoblastic shell. Contributors to this sequence are the mother, who not only donates part of her genome but her reproductive tissues, of which the luteal phase decidua is arguably the most important, as proposed by Professor Conrad. Her partner donates part of his genome in his sperm, the associated seminal fluid, which also can pre-immunize the mother and initiate circuits of immunoregulation, important for the success of later pregnancies. T regulatory cells may be activated (Professor Moss), before conception by the same partner, but not after a short first coitus-conception interval, which then increases the risk of preeclampsia.

But the placenta is the key player with its imprinted genome and two circulations. It is nerveless and, in that sense, its own master, a metabolic controller (Dr. Myers), an invader of the maternal placental bed (Professor Cartwright) and enables more remote and very long-term colonization of maternal tissues by immune and possibly other fetal cells (microchimerism – Dr. Gammill) as well as promoting fetal growth and development. Analysis of priceless but rare CVS samples (Professor Conrad) allows the conclusion that poor placentation may be the outcome of a primary defect in endometrial maturation.

He emphasized that an additional important determinant of prepregnancy and early pregnancy responses is inflammation. Recent research points to the importance of decidual inflammation in promoting progesterone resistance, which predisposes to infertility, early pregnancy loss and preeclampsia, all on the spectrum of poor placentation. Insulin resistance and obesity are associated with systemic inflammation (also called metaflammation), a general cause of metabolic ill health. In general, inflammation is the biological response to ‘danger’. Metaflammation is a non-resolving sterile inflammation and is, like senescence, an endogenous source of danger, which occurs mostly in the context of dietary surfeit. It should be remembered that food intake causes a postprandial, systemic inflammatory response, of which the magnitude depends on the volume and quality of the meal. Whereas inflammation is normally described in terms of its expression in leukocytes and endothelium it is relevant that it can originate in, or impact on, other cells through other stress responses, such as oxidative or endoplasmic reticulum stress. So better pregnancy health is likely to be achievable by healthier diets (Professor Steegers-Theunissen), starting before conception. The importance of diet extends to intrauterine programming of the fetus, the pre-implantation embryo being vulnerable to nutritional, biochemical and physical stress [3]. In terms of prepregnancy care, the prospective father should not be forgotten. For example, paternal body mass has a greater impact than the mother's on body fat and metabolic measures in prepubertal girls [4] and paternal smoking has been associated with increased increase risk of cancer in offspring [5].

A major concern is that poor placentation involves largely early onset preeclampsia, which is only about 20% of all cases of preeclampsia. It is likely that early pregnancy programming may influence events that are not manifest until late pregnancy. There is increasing evidence for example that late-onset preeclampsia also results from syncytiotrophoblast stress but limited to the end of pregnancy and secondary to changes associated with the placenta outgrowing its circulation [6] or becoming senescent or both. The mechanism generating the maternal syndrome (syncytiotrophoblast stress) is similar to that of early onset preeclampsia but the underlying causes are different.

Another concern is that poor placentation is expressed mainly in the uteroplacental circulation. Maldevelopment of the fetoplacental circulation, which in its extreme form is expressed as distal villous hypoplasia, is a pathology that seems to be only distantly related to pool placentation but also causes fetal growth restriction.

The emerging imperative is the need for more sophisticated prepregnancy and early pregnancy care, for good diagnostic markers of successful decidualization (possibly glycodelin) and of very early placental (trophoblast) growth (for example at 4–6 weeks by menstrual dates).

## 5 Recommendations

The presentations and subsequent discussions strongly supported the research community pushing back concepts guiding our research and our research strategies to address prepregnancy and very early pregnancy changes. Information from these studies provides insights but also special challenges. The following recommendations seek to exploit these opportunities and suggest how to address the special challenges.

### 5.1 Epidemiological associations should be explored mechanistically

There are strong relationships between diseases such as chronic hypertension, diabetes and renal disease and behavioral factors including smoking, obesity, diet and exercise as well as race and socioeconomic status with preeclampsia. Studies of obesity have compared obese women who do or do not develop preeclampsia to decipher mechanisms. In view of the complexities and heterogeneity of other predisposing factors, large studies are necessary to address similar relationships. Also interactions of these several prepregnancy risks should be addressed in collaborative, large scale, epidemiological studies.

### 5.2 The relationship of periconceptional behavior and diet with adverse pregnancy outcomes is ready to be explored with intervention studies

Diet, both its quality and amount, and behavioral factors are associated with adverse outcomes. Preliminary studies indicate that intervention in a finite time period (14 weeks before to 10 weeks after conception) can influence these outcomes. This is relevant in both low and high resource settings.

### 5.3 Collaborative, innovative approaches are needed to facilitate studies of the relevance of prepregnancy changes in maternal decidua and early pregnancy interactions of maternal and fetal tissues

The use of residual chorionic villus samples has provided exciting hints of the importance of early maternal and fetal interactions. Studies of early blastocyst indicate that structures and functions that determine pregnancy outcome are established in very early gestation. The immunological interactions of mother and fetus are now better understood. However, chorionic villus samples are now less available as genetic studies increasingly utilize non-invasive approaches. It is important that the research uses of this scarce tissue are maximized through the formation of a chorionic villous sample (CVS) registry. The findings from studies of these tissues will be strengthened by ensuring that they are correlated with measures of circulating markers of appropriate decidualization and of maternal fetal interactions. Markers (predictors) of pregnancy outcome have not been useful in early pregnancy to modify clinical care but may be sufficient to categorize tissues from early pregnancy terminations for *in vitro* studies. Thus far, Doppler velocimetry has been used for this purpose but other biomarkers and combinations of biomarkers should also be explored. The feasibility of using maternal and fetal cells in cervical mucous or fetal tissue in circulating placental microvessicles is also worth exploration.

### 5.4 The relationship of maternal fetal microchimerism to normal and abnormal pregnancy outcomes needs to be studied before during and after pregnancy

The implications of the presence of her mother's and fetus's cells in a pregnant woman are only beginning to be appreciated. Emerging information suggests these relationships are at least quantitatively different in relation to adverse pregnancy outcomes. Microchimerism seems to be a carefully regulated immunological event that has implications that extend beyond pregnancy. There is little if any information about the implications of this phenomenon prior to pregnancy.

### 5.5 We recommend that these studies should be carried out in both high and low resource settings

Cultural, socioeconomic, environmental, nutritional and racial differences in low and high resource settings, influence pregnancy outcomes. Studies to understand the impact of early pregnancy events in low resource settings, where maternal and infant morbidity and mortality is highest, mandate that these be performed in these settings.

Increasing attention to understanding maternal fetal interactions before pregnancy and in very early pregnancy will yield new understanding of adverse pregnancy outcomes. The success of early interventions such as folic acid is an early proof of the principle that these insights could guide useful preventive/therapeutic strategies.
